# A motion characteristics modeled angular position sensor by nonlinear transfer of differential capacitance for miniaturized scanning mirrors

**DOI:** 10.1038/s41378-023-00619-8

**Published:** 2023-11-24

**Authors:** Songtao Liu, Gaofei Zhang, Lingyun Zhang, Junya Wang, Minghao Gong, Zheng You

**Affiliations:** 1 Department of Precision Instrument, Tsinghua University, Beijing, 100084 China; 2 Key Laboratory of Smart Microsystem (Tsinghua University), Ministry of Education, Beijing, 100084 China; 3 State Key Laboratory of Precision Measurement Technology and Instruments, Tsinghua University, Beijing, 100084 China; 4 School of Mechanical Science & Engineer, Huazhong University of Science and Technology, Wuhan, 430074 China

**Keywords:** Sensors, Electrical and electronic engineering

## Abstract

In this paper, an angular position sensor (APS) designed for a resonant miniaturized scanning mirror (M-SM) is presented. The APS operates based on the principle of differential variable capacitance, significantly expanding the detectable bandwidth from a few hertz to several kilohertz. By modeling the motion characteristics, the sampling rates of the biaxial scanning angles are 1473.6 times and 539.4 times higher than those of conventional sensors. Initially, the motion characteristics model is presented as a simple harmonic motion, converting sampled capacitance into continuous capacitance. Subsequently, the nonparallel state of the M-SM and sensor is transformed into a parallel state through the space coordinate system transformation. Furthermore, a 2D nonlinear angle transfer function is developed to convert the differential capacitance into an angle, thereby mitigating the nonlinear errors resulting from large angles. Achieving an accuracy better than 0.014°, the measuring range expands from ±0.5729° (±10 mrad) to ±5.026° ( ± 87 mrad). Additionally, the capturing mode and tracking mode are proposed to monitor real-time angular changes of the M-SM with an accuracy of 0.017°. High-precision APSs have enhanced beam pointing accuracy and resolution and can thereby be used to advance the development of laser components, including light detection and ranging (LiDAR).

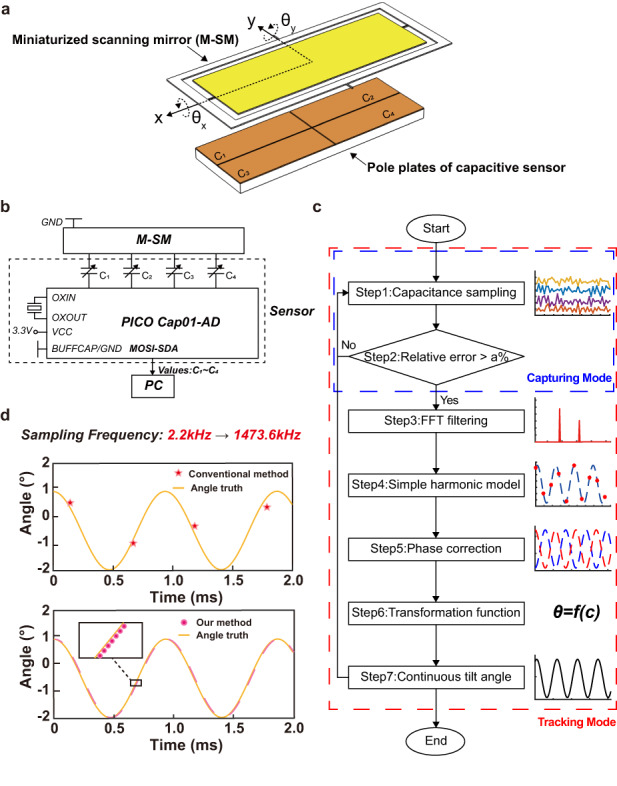

## Introduction

Angular position sensors (APSs) are in high demand across various industrial, automotive, and robotic applications^1^. Recent advancements in micro-electromechanical system scanning mirror (MEMS-SM)-based LiDAR have resulted in new APSs that are capable of measuring the out-of-plane scanning angle. APSs for MEMS-SM have been developed based on piezoresistivity^2–8^, piezoelectricity^9–11^, electromagnetic induction^12–15^, active detection^16–21^, etc.

The piezoresistive APS features simple manufacturing processes and high integration^2,3^. The angle estimation algorithm based on the unscented Kalman filter (UKF) was employed to reduce noise^4^. The piezoresistive coefficient was estimated through master-mode frequency response function (FRF) curve analysis^5^, and closed-loop control using Wheatstone bridges and diffusion piezoresistive sensors was implemented^6^. A calibrated scale is utilized to accurately calibrate the voltage signal of the piezoresistive sensor, ensuring a precision of 0.5° or less^7^. A temperature compensation scheme is proposed to accommodate temperature noise^8^.

The piezoelectric APS is fabricated along with the aluminum nitride (AIN) driving film, and a maximum shape error of 25% may be caused^9^. The driving voltage of the aluminum scandium nitrogen (AlScN) film is calibrated using a position sensitive detector (PSD) to determine the angle; however, motion nonlinearity can lead to calibration failure^10^. An accuracy of less than 0.1° is achieved by precise position feedback control^11^.

The electromagnetic APS comprises an integrated permanent magnet and an energized coil on the MEMS-SM. The accuracy of 0.067° is obtained by establishing the equations of the induced electromotive force and scanning angle^12^. Accuracy is further improved by optimizing the coil position^13^. Typically, the generated induced electromotive force is extremely weak and requires amplification by several hundred times for detection^14^. Without utilizing the electromagnetic driving principle, additional coils and permanent magnets would need to be assembled, resulting in increased volume loads within the system^15^.

The active detection APS comprises a VCSEL and three photodiodes (PDs) that receive optical power proportional to the small scanning angle of 5°^16^. The scanning angle is determined by measuring the differential optical voltage of the quadrant photodetectors (QPDs), achieving an accuracy of 0.1°^17^. Laser diodes, QPDs, and readout circuits can be integrated onto a single chip^18^. The configuration incorporating nine PDs with high-resolution optical sensors and calculations ensured a goniometric accuracy better than 0.1°^19^. The PD was replaced with a PSD, reducing the measurement uncertainty to approximately 0.026°. Furthermore, the main sources of error were analyzed^20^. The size of the PSD sensor and the laser module significantly exceeds that of MEMS-SM, resulting in increased volume load^21^.

However, the constrained mirror size and limited angular measurement accuracy of MEMS-SM limit the ability of LIDAR to detect long-range targets. Non-silicon-based miniaturized scanning mirrors (M-SMs) have been proposed for obtaining larger apertures, and APS schemes with higher accuracy have been explored.

Differential capacitors provide excellent accuracy performance^22,23^, and associated multi-segment electrodes are commonly employed in in-plane angle measurements, characterized by uniform spacing between the capacitive electrodes. The straightforwardness of this principle has spurred significant research endeavors, particularly in applications such as measuring the rotational angles of motors or gyroscopes^1,24,25^. Nonetheless, when the capacitor pole-plate spacing dynamically changes with the angle, the introduction of numerous nonlinear calculation terms complicates the theoretical model describing the relationship between the angle and capacitance. Consequently, this situation necessitates the development of a novel and more precise capacitance model for APSs. Additionally, the challenge of low data update rates is a critical issue in the context of capacitance angle measurements^26–34^.

The conventional capacitive APS featured multiple electrodes and was designed to measure the scanning angle of a charged flat plate with a static relative error below 3.7%^22^. However, static calibration is utilized to convert readout circuit voltages into scanning angles, and the absence of a theoretical model is notable. Moreover, the sampling rate of the common capacitive readout circuit leads to insufficient angular resolution for actuators with high-frequency motion (kHz). A novel bridge circuit configuration was developed to enhance the accuracy of detecting the static displacement and scanning angle, achieving a relative error of 0.001° and a measuring range of ±0.5729° (±10 mrad)^23^. However, the scanning angle and differential capacitance exhibit linearity at small angles, enabling the accurate measurement of a scanning angle of ±10 mrad under the assumption of linearity. Nevertheless, the nonlinear error progressively increases with larger scanning angles.

Capacitive APSs are more accurate and are well suited for long-range LiDAR applications. However, it is important to consider certain shortcomings, including slow data update rates and nonlinear errors.

Some interpolative extrapolation methods can improve the data update rates by inferring the angle information between the sampling points, such as the piecewise cubic Hermite interpolating polynomial (Pchip)^24^, modified Akima (Makima) method^25^, B-spline method^26^, and radial basis function (RBF) method^27^. Regarding their respective characteristics, the Pchip method exhibits a tendency to flatten out around local extremes, the Makima method improves upon oscillatory behaviors inherent to interpolation curves, the B-spline method relies on B-spline functions for data interpolation, and the RBF method adapts the kernel function according to the interpolated object, thereby enhancing interpolation accuracy. Notably, these methods are generalized approaches that primarily rely on available sampling points to accomplish interpolation tasks. This leads to a high degree of randomness in the extrapolation between sampling points. Conversely, an explicit model detailing the motion characteristics of the M-SM can effectively mitigate this randomness, thereby leading to a substantial improvement in the interpolation accuracy^28^.

Analytical modeling of nonparallel pole plate capacitors provides a potential solution to address nonlinear errors^29–32^. The first estimation model for nonparallel pole-plate capacitors with small angles is developed^29^, enabling the calculation of capacitance between two rectangular pole plates in 2D at any position. The nonlinear response resulting from the scanning angle of the pole plate can be used to estimate the positional attitude error^30^. A flexible nonparallel polar plate with the center of rotation at the elbow joint is utilized to detect the angle of the elbow joint^31^. The conformal mapping-based 3D capacitance modeling of nonparallel plates is proposed^32^, with the center of rotation located at the intersection of the out-of-plane region of the 3D nonparallel polar plates and the model capable of calculating only the 1D angle. Model building is challenging for the M-SM, which is one of the nonparallel pole-plate capacitors that scans in 2D around its center of mass.

In this paper, a capacitive APS for M-SM is developed with a primary focus on angular accuracy to enhance the distance measuring range of LIDAR. The independent configuration of four-quadrant differential capacitors provides excellent environmental robustness because differential calculations effectively mitigate external electromagnetic noise. To overcome the limited data update rate of conventional capacitive APSs, the proposed M-SM motion characteristics model ensures continuous real-time differential capacitance, resulting in a significant increase in the data update rate by several hundred times compared to relying solely on capacitance-to-digital conversion (CTDC) circuits. The application of a nonlinear transfer equation, followed by fast Fourier transform (FFT) frequency division, converts differential capacitance into a biaxial scanning angle, expanding the measuring range from ±0.5729° to ±5.026° while ensuring an accuracy better than 0.014°. Two operating modes are proposed to accommodate scenarios with varying angular change rates, achieving tracking accuracy better than 0.017°.

## Results and experiments

### The proposed APS system

**Fig. 1:**
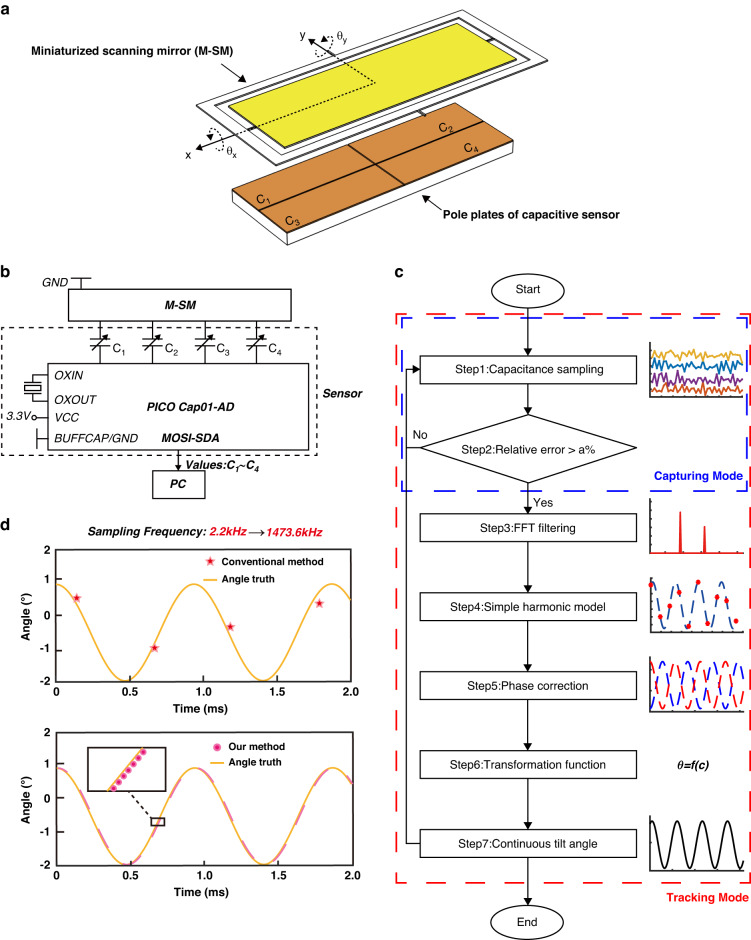
The principle of the data processing algorithm. **a** The APS comprises four capacitors and a CTDC circuit (shown in (**b**)). The four capacitors are formed by a dual-axis M-SM (yellow plate) serving as the common grounded pole plate and four independent metal pole plates (brown plate). **b** The circuit system diagram consists of the grounded M-SM, four capacitors, PICO Cap01-AD CTDC, and a personal computer. **c** Flowchart of the proposed data processing algorithm, including two modes and seven steps. **d** Scanning angle sampling data before and after applying the proposed data processing method

The proposed sensor system architecture consists of three main components. The yellow plate represents the M-SM, which rotates around the *x-* and *y*-axes with scanning angles of *θ*_x_ and *θ*_y_, respectively (Fig. 1a). Beneath the M-SM lies an APS consisting of four conductive metal pole plates (colored brown) and a CTDC circuit. The M-SM acts as the upper pole plate of the capacitor. The four plates are distributed in Quadrants 1 to 4, forming Capacitors C1 to C4 with the grounded M-SM. Figure 1b presents a schematic diagram of the CTDC circuit. The M-SM is connected to the circuit ground, while the 4-quadrant metal lower pole plates of the sensor are connected to the PICO Cap01-AD circuit. The CTDC circuit outputs four real-time capacitances asynchronously to a personal computer, which executes the software-side data processing algorithm (DPA). The DPA consists of 7 steps, taking the capacitance sampling data as the input and producing real-time continuous capacitance data as the output (Fig. 1c). Through digital sampling of real-time continuous capacitance data, the sampling performance is significantly enhanced, increasing from 2.2 kHz to a maximum of 1473.6 kHz. This improvement effectively enhances the angular resolution of the sensor, as shown in Fig. 1d.

**Fig. 2:**
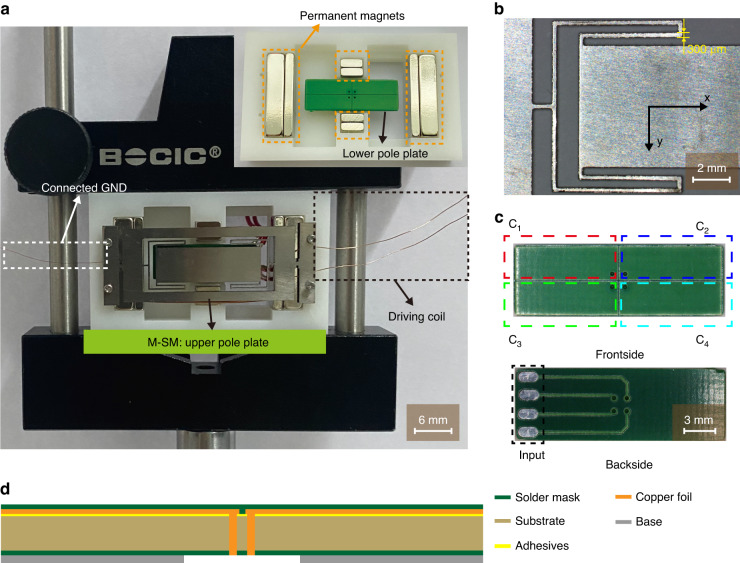
Experimental configuration. **a** The M-SM system comprises a Ti-based M-SM, a driving coil, a driving magnetic circuit, and an encapsulated housing. **b** Micrograph of a half-side M-SM with a minimum line width of 300 μm for a cantilever beam. **c** Front and backside views of the 4-quadrant metal pole plates. **d** Side view of the metal pole plates based on printed circuit board technology

The fabricated M-SM system is shown in Fig. 2a, with the proposed capacitive APS assembled beneath the M-SM. The experimentally measured resonant frequencies are 892.0 Hz (*x*-axis) and 959.2 Hz (*y*-axis). To achieve maximum static capacitance, the M-SM and sensor are assembled face-to-face, with a distance greater than the maximum displacement of the M-SM in the z-direction to prevent collisions. The M-SM, fabricated using a non-silicon based conductive material (Ti-6Al-4V), as shown in Fig. 2b, can be ground directly due to the excellent conductivity of the alloy, eliminating the need for additional process steps. The performance of the APS is experimentally characterized in subsequent subsections. The processing method of the sensor is similar to that of a printed circuit board (PCB), as illustrated in Fig. 2c. Figure 2d depicts the components of the sensor: the FR-4 solder mask functions as a protective layer, the copper foil forms the conductive pole plate, the EVA adhesives connect the copper to the protective layer, the ERGCL substrate constitutes the sensor body with perforations at the bottom for energization, and the 3D printed base serves as the housing for the entire system.

Capacitors *C*_1_ - *C*_4_ are combined into Series Capacitor Banks (*SCB*_1_ - *SCB*_4_) to represent *θ*_x_ and *θ*_y_, as indicated in Table 1. The value of *θ*_x_ is determined by the difference capacitance (*DC*_x_) between *SCB*_1_ and *SCB*_2_, while *θ*_y_ is determined by the difference capacitance (*DC*_y_) between *SCB*_3_ and *SCB*_4_. The components obtained from *C*_1_ - *C*_4_ after an inverse FFT at the resonant frequency are represented by *C*_1x_ - *C*_4x_ and *C*_1y_ - *C*_4y_. The capacitance of the Series Capacitor Bank (*SCB*) is the sum of individual capacitors *C*_1_ - *C*_4_.

**Table 1:** Scanning angles and corresponding series capacitor banks

Scanning angle	Type	Note
*θ*_x_	*SCB*_1_	*C*_1_ and *C*_2_ (*C*_1x_ and *C*_2x_)
	*SCB*_2_	*C*_3_ and *C*_4_ (*C*_3x_ and *C*_4x_)
*θ*_y_	*SCB*_3_	*C*_1_ and *C*_3_ (*C*_1y_ and *C*_3y_)
	*SCB*_4_	*C*_2_ and *C*_4_ (*C*_2y_ and *C*_4y_)

In the 1D scanning mode, as one half of the M-SM moves closer to the two conductive metal plates below it, the corresponding capacitances increase. Similarly, the other half moves away from the corresponding pole plates, resulting in a decrease in the two capacitances.

**Fig. 3:**
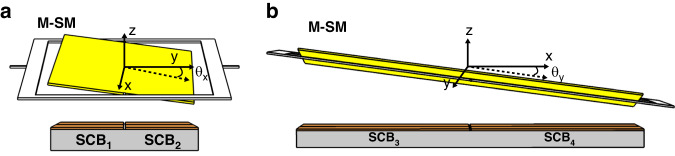
M-SM in the scanning state. **a** Simplified diagram of the M-SM scanned around the *x*-axis. **b** Simplified diagram of the M-SM scanned around the y-axis

For instance, when the M-SM scans counterclockwise around the *x*-axis (Fig. 3a), *SCB*_1_ increases while *SCB*_2_ decreases. Similarly, when the M-SM scans counterclockwise around the *y*-axis (Fig. 3b), *SCB*_3_ increases while *SCB*_4_ decreases.

In the 2D scanning mode, due to the different *x*- and *y-*axis resonant frequencies, *C*_1x_ - *C*_4x_ and *C*_1y_ - *C*_4y_ are utilized to describe the biaxial capacitance components retrieved through FFT.

Additionally, the capacitance of the M-SM exhibits weak variation, and it is necessary to consider electromagnetic interference. Electromagnetic interference primarily arises from high-frequency driving signals, active circuits, and environmental factors, which can be mitigated through FFT filtering in the data processing algorithm. The common ground capacitive circuit is less complex than the conventional one-to-one CTDC, where the M-SM upper plate is divided into four quadrants, with each quadrant independently facing the lower plate to form *C*_1_ - *C*_4_.

### Data processing algorithm

Prior to executing the data processing algorithm, the 4-channel capacitors are asynchronously sampled multiple times and used as the initial group in Step 1 (Fig. 1c).

In the first step, the CTDC circuit samples them once more and determines whether the relative error between this group and the initial group surpasses a specific threshold, denoted as *a*%. This threshold indicates the degree to which the M-SM deviates from the initial scanning angle.

If the threshold is surpassed, the system enters the capturing mode to execute the remaining five steps and obtain the most recent real-time angle. If the threshold is not surpassed, the system continues to operate in the tracking mode, and Steps 1 and 2 are repeated. The primary objective is to detect any deviations of *θ* from the preset and enable integration with potential closed-loop control algorithms in the future.

Simultaneously, external electromagnetic interference and internal high-frequency interference within the CTDC circuit affect the sampled differential capacitance values, leading to measurement errors.

In Step 3 of our analysis, we employed an a priori-bandwidth-based FFT filtering algorithm to effectively mitigate interference. In this context, the term “a priori bandwidth” refers to a narrow frequency range centered around the driving frequency of the MEMS-SM. The angular or capacitive feedback frequency of the MEMS-SM aligns precisely with the driving frequency, resulting in a nondestructive feedback signal that exhibits a single-peak pattern in the amplitude-frequency characteristic curve. In cases involving two-dimensional motions, this pattern may manifest as a bimodal pattern due to differences in the driving frequencies for the two dimensions. Interference noise, on the other hand, is randomly distributed across the frequency band and superimposed onto the single peak of the lossless feedback signal. When we configure the filter passband to match the a priori bandwidth, the nearly lossless angular or capacitive feedback signals are retained, while a substantial portion of the interference noise is simultaneously filtered out. This approach effectively preserves the integrity of the desired signals while suppressing the unwanted noise components.

Step 4 involves modeling the filtered sampled capacitance using motion characteristics to ensure continuity and enable estimation of the unknown capacitance between the sampled points.

In Step 5, inconsistent temporal phases due to the high-frequency vibration of the M-SM during angle sampling are addressed. To rectify this issue, phase correction is employed to align the motion characteristics model with the reference moment in time. Subsequently, the motion characteristics model is differentially calculated based on different axis types, yielding the *DC* model.

Step 6 involves calculating the maximum differential capacitance within the *DC* model, which is subsequently adjusted to determine the maximum scanning angle using the nonlinear scanning angle transfer function. In Step 7, which is the conclusion of this process, the continuous scanning angle is obtained. Compared to converting all sampled points into scanning angles, transferring the maximum scanning angle conserves computational resources and enhances real-time performance.

### Motion characteristics model and phase correction

**Fig. 4:**
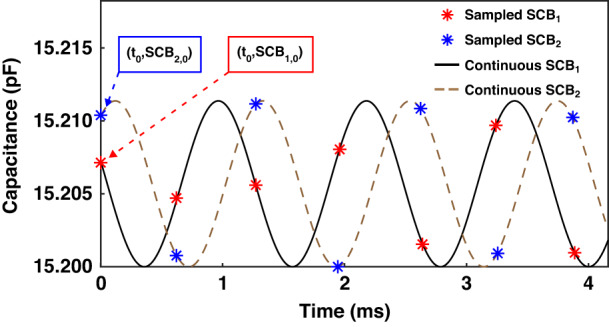
Principle of the motion characteristics model. The real-time model of the differential capacitive simple harmonic motion characteristics along the *x*-axis

We propose a real-time motion characteristics model for describing the simple harmonic motion characteristics of differential capacitive systems (Fig. 4). This model effectively converts discrete capacitance samples into continuous curves.

The discreteness of the sampling originates from the operating frequency of the CTDC circuit, and real-time refers to the time alignment between each capacitance sample and its corresponding sampling moment. Conventional capacitive APS suffers from limited real-time performance due to the low sampling frequency of the CTDC circuit (~kHz), which is comparable to the scanning frequency of the M-SM. As a result, they are unsuitable for LiDAR applications that demand high frame rates and dense point clouds. Applying a continuous simple harmonic driving voltage to the coil induces an approximate harmonic response in the M-SM’s scanning when the frequency is in proximity to its resonance region. The differential capacitance exhibits simple harmonic motion due to the inherent relationship between the scanning angle and the differential capacitance. Transforming discrete differential capacitance into a continuous representation can be accomplished by fitting a simple harmonic curve to the discrete data points.

The motion characteristics model is synchronized with the time record of the sampled differential capacitance. The differential capacitive motion characteristics model can be expressed as:

1 $$D{C}_{x}=D{C}_{x\max }\,\sin ({\omega }_{x}t-{\varphi }_{x})$$

*DC*_xmax_ represents the maximum differential capacitance achievable by solving the motion model over a single period. *ω*_x_ represents the angular resonant frequency of the M-SM scanned around the *x*-axis, with *ω*_x_ = 2π*f*_x_ and *f*_x_ being the resonant frequency of the *x*-axis. *φ*_x_ represents the phase of the scanning angle that can be determined through curve fitting.

In addition:

2 $$D{C}_{0x}=D{C}_{x\max }\,\sin ({\omega }_{x}{t}_{0}-{\varphi }_{x})$$

*DC*_0x_ denotes the initial differential capacitance at moment *t*_0_. Similarly, the differential capacitance of the M-SM at any moment, including the initial moment, about the *y*-axis can be expressed as:

3 $$D{C}_{y}=D{C}_{y\max }\,\sin ({\omega }_{y}t-{\varphi }_{y})$$

4 $$D{C}_{0y}=D{C}_{y\max }\,\sin ({\omega }_{y}{t}_{0}-{\varphi }_{y})$$

By substituting the calculated maximum differential combination capacitance, resonant frequency, and phase into the capacitive motion characteristics model, we can determine the values of *DC*_x_ and *DC*_y_ at any given moment, including *t*_0_. In this way, it is possible to obtain sampled capacitance values for extremely small time intervals, effectively providing an approximation of an infinite sampling frequency.

Asynchronous sampling introduces sampling time variations for *C*_1_ - *C*_4_, which are intended to capture a specific angle during simultaneous sampling. However, these variations lead to asynchronous sampling errors. To mitigate these errors, it is necessary to perform phase correction based on predefined motion rules. Phase correction comprises relative phase correction and absolute phase correction. Relative phase correction eliminates phase errors between the *SCB*s but forfeits the original time record. Absolute phase correction synchronizes the phase of *SCB*s with the original time record, thereby ensuring real-time accuracy of the motion model curve.

**Fig. 5:**
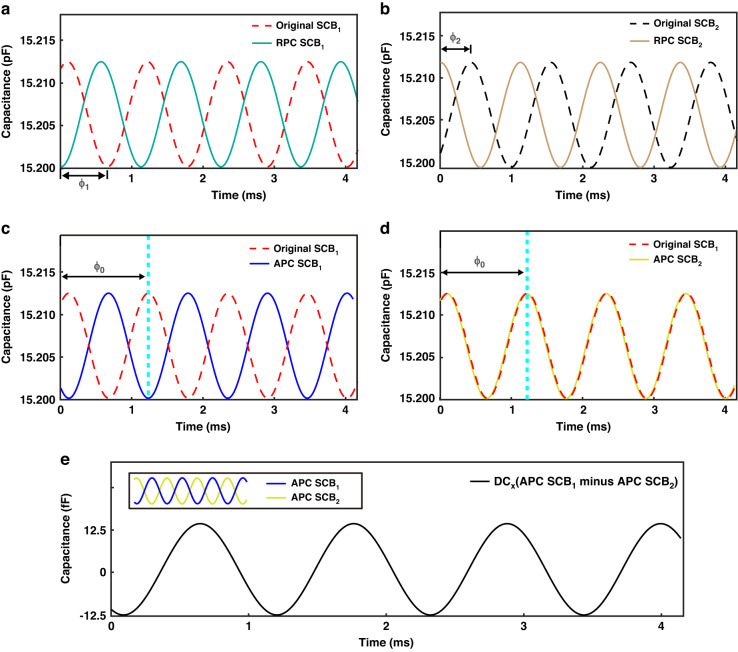
Principle of the phase correction. **a** Relative phase correction of *SCB*_1_. **b** Relative phase correction of *SCB*_2_. **c** Absolute phase correction of *SCB*_1_. **d** Absolute phase correction of *SCB*_2_. **e** The *DC*_x_ model characteristics curve is obtained by differencing *SCB*_1_ and *SCB*_2_ after phase correction

For the *x*-axis scanning example, relative phase correction is feasible since *SCB*_1_ and *SCB*_2_ exhibit precisely opposite phases during mechanical vibration. When *SCB*_1_ reaches its minimum, *SCB*_2_ reaches its maximum. The phases *φ*_1_ and *φ*_2_, corresponding to the valley of *SCB*_1_ and the peak of *SCB*_2_, are calculated and added to the phase terms (phase advance) of the *SCB*_1_ and *SCB*_2_ motion models. This yields the relative-phase corrected *SCB*_1_ and *SCB*_2_ (Fig. 5a, b). After applying relative phase correction, *SCB*_1_ is positioned at the valley, and *SCB*_2_ is positioned at the peak at the initial moment. Regarding absolute phase correction (Fig. 5c, d), the relative-phase corrected *SCB*_1_ and *SCB*_2_ are shifted to the opposite or aligned with the original *SCB*_1_. The phase *φ*_0_ of the second peak of the original *SCB*_1_ is determined. Subsequently, *φ*_0_ is subtracted from the phase terms (phase delay) of the *SCB*_1_ and *SCB*_2_ motion models, resulting in the differential capacitance that is exactly opposite or identical to the original *SCB*_1_.

Finally, a nonlinear real-time *DC*_x_ motion characteristics model is obtained by subtracting *SCB*_1_ from *SCB*_2_ (Fig. 5e). Similarly, *DC*_y_ can be obtained following the same steps as *DC*_x_.

### Nonlinear angle transformation function

A nonlinear angle transformation function is employed to convert the amplitudes of the *DC*_x_ and *DC*_y_ motion models obtained from the nonparallel pole-plate capacitors into *θ*_xmax_ and *θ*_ymax_, respectively. This conversion allows for the construction of a complete 2D scanning angle motion characteristics model.

The capacitance calculation method for parallel pole-plate capacitors is well known, as it is based on the proportional relationship between capacitance and the projected area and the inverse relationship with the distance between pole plates. However, the challenge lies in transforming the nonparallel pole plates into a parallel configuration.

To achieve parallelism between the two pole plates, we employ a series of consecutive coordinate system transformations that convert the real coordinate system into an imaginary coordinate system^25^.

Then, the conventional nonparallel plate capacitance calculation method is employed to derive the transfer function. The key differences between the proposed and conventional methods lie in the position of the rotation center and the dimensional representation of the scanning angle. Typically, the rotation center is located at the intersection of the extensions of the nonparallel pole plates, allowing for modeling of a 1D scanning of the pole plates. However, in our approach, the scanning center is situated at the midpoint of the M-SM. Combining the immobile point constraint located at the midpoint with the conventional nonparallel plate capacitance calculation method, a novel nonlinear angle transformation function for M-SM is proposed.

**Fig. 6:**
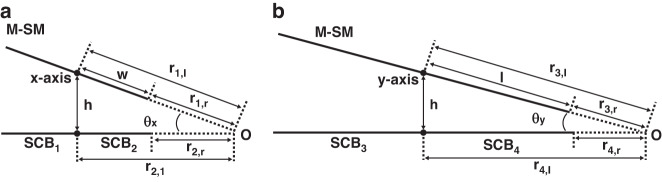
Parameters of the non-linear angle transformation function. **a** Explanation of the geometric parameters of the M-SM for the nonlinear angle transformation function along the *x*-axis. **b** Explanation of the geometric parameters of the M-SM for the nonlinear angle transformation function along the *y*-axis

In Fig. 6a, b, the extension lines of the upper and lower pole plates intersect at Point O. The half-width w of the M-SM matches the width of any individual four-quadrant polar plate, and the distance *h* represents the separation between the scanning center of the M-SM and the four-quadrant pole plates. The distances from the *x*-axis/boundary of the M-SM to Point O are denoted as *r*_1,l_ and *r*_1,r_, while the distances from the center/boundary of the four-quadrant pole plates to Point O are represented as *r*_2,l_ and *r*_2,r_. Subsequently, a series of consecutive coordinate system transformations is executed.

Equations (5, 6) depict the 1D nonlinear angle transformation functions around the *x*-axis derived from the utilization of nonparallel pole-plate capacitors:

5 $$D{C}_{x}={\varepsilon }_{0}\left({\varDelta }_{x}\frac{S({k}_{in,x,i}({\theta }_{x}))}{d({k}_{in,x,i}({\theta }_{x}))}+{\varDelta }_{x}\frac{S({k}_{out,x,i}({\theta }_{x}))}{d({k}_{out,x,i}({\theta }_{x}))}\right)$$

6 $${k}_{in,x,i}({\theta }_{x})={\left(\frac{{({r}_{1,i}+W)}^{\frac{\pi }{{\theta }_{x}}}-{r}_{1,i}^{\frac{\pi }{{\theta }_{x}}}}{{({r}_{1,i}+W)}^{\frac{\pi }{{\theta }_{x}}}+{r}_{2,i}^{\frac{\pi }{{\theta }_{x}}}}\right)}^{\frac{1}{2}}\cdot {\left(\frac{{({r}_{2,i}+W)}^{\frac{\pi }{{\theta }_{x}}}-{r}_{2,i}^{\frac{\pi }{{\theta }_{x}}}}{{({r}_{2,i}+W)}^{\frac{\pi }{{\theta }_{x}}}+{r}_{1,i}^{\frac{\pi }{{\theta }_{x}}}}\right)}^{\frac{1}{2}}$$

Here, *ɛ*_0_ represents the free-space permittivity, and

7 $${\varDelta }_{x}(\bullet )=\frac{S(i=r)}{d(i=r)}-\frac{S(i=l)}{d(i=l)}$$

denotes the subtraction of the function “●” with *i* being either *r* or *l*, corresponding to *SCB*_1_ and *SCB*_2_, respectively.

8 $$S(\cdot )/d(\cdot )=-\,\log (\cdot )/\pi$$

Equation (8) represents the evolving equation for calculating the capacitance of a parallel plate capacitor in an imaginary coordinate system. *k*_in,x,i_(*θ*_x_) and *k*_out,x,i_(*θ*_x_) represent the values derived from the imaginary coordinate system inside and outside the pole plate, respectively, when the scanning angle of the pole plate is *θ*_x_.

9 $${k}_{out,x,i}({\theta }_{x})={{k}_{in,x,i}({\theta }_{x})|}_{{\theta }_{x}\to 2\pi -{\theta }_{x}}$$

10 $${r}_{1,r}=\frac{h}{\sin {\theta }_{x}}-W$$

11 $${r}_{2,r}=\frac{h}{\tan {\theta }_{x}}-W$$

12 $${r}_{1,l}=\frac{h}{\sin {\theta }_{x}}$$

13 $${r}_{2,l}=\frac{h}{\tan {\theta }_{x}}$$

Equations (9, 13) are established rules that are used to enforce a specific distance *h* between the central axis of the M-SM and the pole plates.

Equations (7, 8) present the 1D nonlinear angle transformation functions for the *y*-axis after defining the rotation rules *r*_3_ and *r*_4_.

14 $$D{C}_{y}={\varepsilon }_{0}\left({\varDelta }_{y}\frac{S({k}_{in,y,i}({\theta }_{y}))}{d({k}_{in,y,i}({\theta }_{y}))}+{\varDelta }_{y}\frac{S({k}_{out,y,i}({\theta }_{y}))}{d({k}_{out,y,i}({\theta }_{y}))}\right)$$

15 $${k}_{in,y,i}({\theta }_{y})={\left(\frac{{({r}_{3,i}+l)}^{\frac{\pi }{{\theta }_{y}}}-{r}_{3,i}^{\frac{\pi }{{\theta }_{y}}}}{{({r}_{3,i}+l)}^{\frac{\pi }{{\theta }_{y}}}+{r}_{4,i}^{\frac{\pi }{{\theta }_{y}}}}\right)}^{\frac{1}{2}}\cdot {\left(\frac{{({r}_{4,i}+l)}^{\frac{\pi }{{\theta }_{y}}}-{r}_{4,i}^{\frac{\pi }{{\theta }_{y}}}}{{({r}_{4,i}+l)}^{\frac{\pi }{{\theta }_{y}}}+{r}_{3,i}^{\frac{\pi }{{\theta }_{y}}}}\right)}^{\frac{1}{2}}$$

Substituting *DC*_xmax_ and *DC*_ymax_ into Eqs. (5, 7) yields *θ*_xmax_ and *θ*_ymax_, respectively. The 2D simple harmonic motion characteristics models can be obtained by replacing *DC*_xmax_ and *DC*_ymax_ with *θ*_xmax_ and *θ*_ymax_, respectively, as shown below:

16 $${\theta }_{x}={\theta }_{x\max }\,\sin ({\omega }_{x}t-{\varphi }_{x})$$

17 $${\theta }_{y}={\theta }_{y\max }\,\sin ({\omega }_{y}t-{\varphi }_{y})$$

It is noteworthy that multiple successive coordinate system transformations allow for different lengths of upper and lower pole plates, as well as the arrangement of any pole plate at any position in the *x*-*y* plane. This flexibility offers significant advantages in the design of angular displacement sensors.

### Specifications

#### Accuracy

**Fig. 7:**
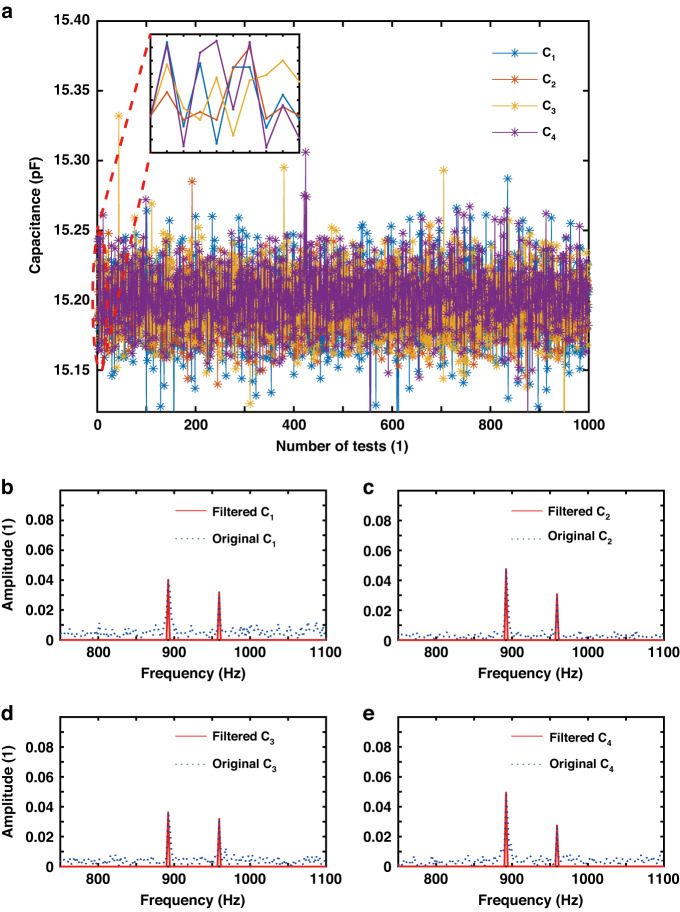
Sampling and filtering. **a** 1000 sets of original four-channel capacitance samples. **b** The amplitude-frequency characteristic curve of *C*_1_ exhibits two peaks that correspond to the resonant frequencies of the M-SM. The larger peak, observed in the *x*-axis at low frequencies, corresponds to an optical scanning angle of 10.4960°, while the smaller peak in the *y*-axis at high frequencies corresponds to an optical scanning angle of 3.9031°. **c** The amplitude-frequency characteristic curve of C_2_. **d** The amplitude-frequency characteristic curve of C_3_. **e** The amplitude-frequency characteristic curve of C_4_

**Fig. 8:**
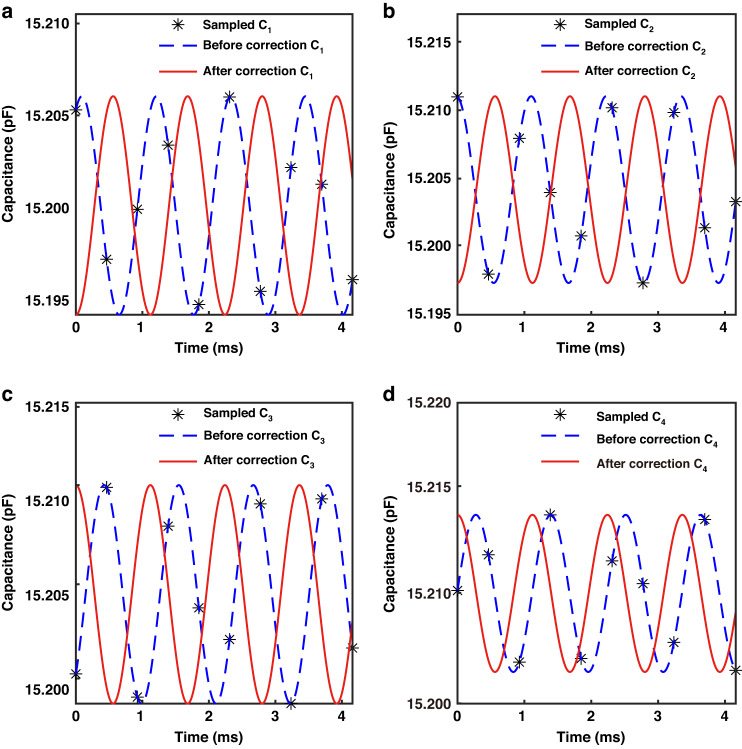
Phase correction of *SCB*_1_ and *SCB*_2_. **a**–**d** The motion characteristics models *C*_1_ to *C*_4_ are derived from the inverse FFT of the low-frequency peaks in the 4 amplitude-frequency characteristic curves. The changes in *C*_1_, *C*_2_ and *C*_3_, *C*_4_ are consistently observed

**Fig. 9:**
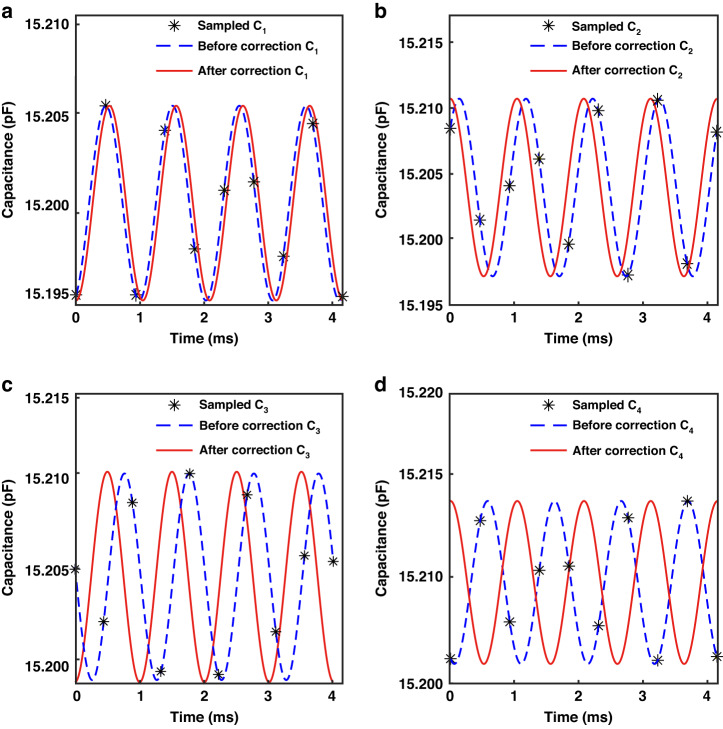
Phase correction of *SCB*_3_ and *SCB*_4_. **a**–**d** The motion characteristics models *C*_1_ to *C*_4_ are derived from the inverse FFT of the low-frequency peaks in the 4 amplitude-frequency characteristic curves. The changes in *C*_1_, *C*_3_ and *C*_2_, *C*_4_ are consistently observed

The CTDC circuit PICO Cap01-AD is used to sample *C*_1_ - *C*_4_, resulting in a total of 1000 × 4 capacitance measurements in this experiment, as depicted in Fig. 7a. The capacitance values of *C*_1_ - *C*_4_ range between 15.15 pF and 15.25 pF. The CTDC circuit has a sampling frequency of 8.96 kHz, while each channel is asynchronously sampled at 2.24 kHz, meeting Nyquist’s sampling criterion by exceeding twice the resonant frequency. The capacitance values were normalized to the interval [0, 1], and frequency spectrum analysis was conducted to derive the amplitude-frequency curve, depicted in Fig. 7b–e. Two peaks can be observed, corresponding to the resonant frequencies of the M-SM. The amplitude of frequencies outside the resonant frequency band is set to zero, effectively filtering out random interference noise, as indicated by the red solid line. By applying inverse FFT, *C*_1_ - *C*_4_ are transformed into *C*_1x_ - *C*_4x_ and *C*_1y_ - *C*_4y_, respectively, as indicated in Figs. 8, 9. The capacitive motion characteristics model of the dual-axis was established by fitting the simple harmonic curve. Subsequently, phase correction is applied using a priori rules of motion. When scanning around the *x*-axis, motion synchronization causes *C*_1_ and *C*_2_ to form *SCB*_1_, shifting its valley to the start point of the time axis, as depicted in Figs. 8a, b. Similarly, *C*_3_ and *C*_4_ form *SCB*_2_, which is shifted to the peak at the initial moment, as illustrated in Figs. 8c, d.

Similarly, when scanned around the *y*-axis, *C*_1_ and *C*_3_ shifted to the valley, while *C*_2_ and *C*_4_ shifted to the peak, as indicated in Fig. 9a–d. Notably, the absolute phase correction was omitted, as it does not affect the accuracy of the sensor.

**Fig. 10:**
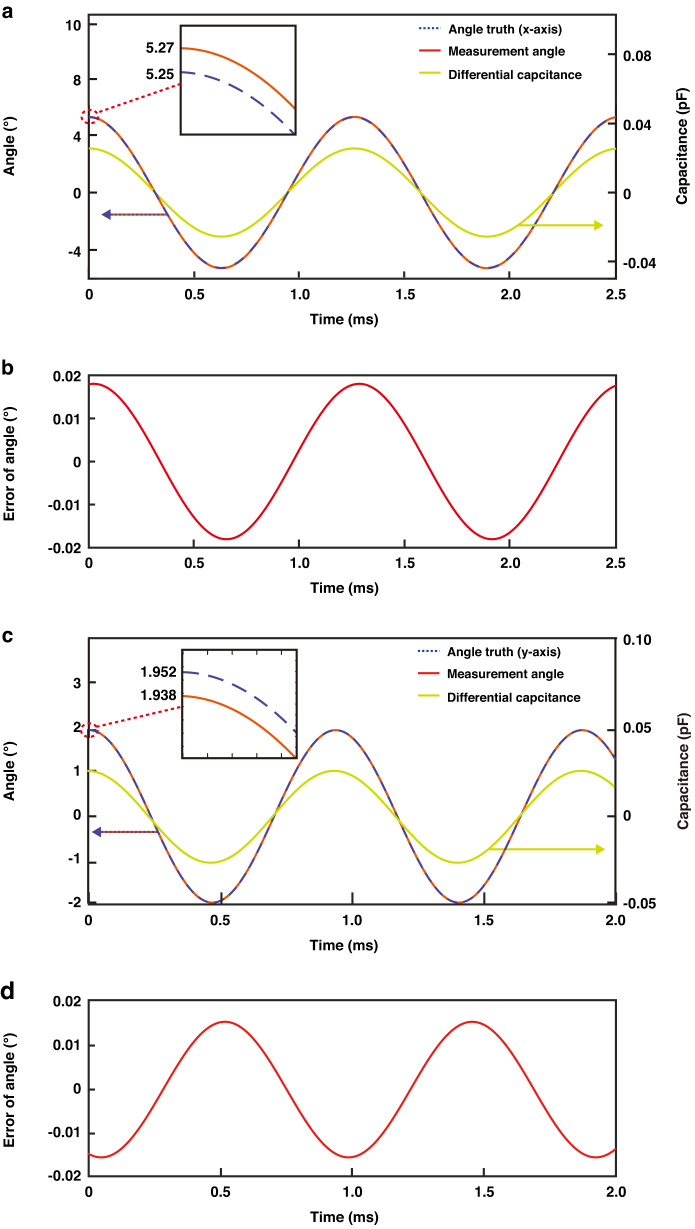
Angle measurement results and errors. **a** Continuous measurement of the optical scanning angle around the *x*-axis. The true optical scanning angle. The differential capacitance on the right scale. **b** Measurement error of the optical scanning angle around the *x*-axis. **c** Continuous measurement of the optical scanning angle around the *y*-axis. The true optical scanning angle. The differential capacitance on the right scale. **d** Measurement error of the optical scanning angle around the *y*-axis

The differential capacitance motion characteristics models *DC*_x_ and *DC*_y_ were established and are represented in Fig. 10a, c. After the amplitude term of the motion model was adjusted using the nonlinear scanning angle transformation function, the scanning angle motion model was established. The ground truth angle is represented by the PSD-based angular measurement, which exhibits lower measurement uncertainty compared to the proposed capacitive APS. The calculation of the measurement uncertainty will be explained in a subsequent article.

Finally, the angular measurement error is shown in Fig. 10b, d. The measurement error increases as the measured optical scanning angle increases. At the initial moment, the maximum measurement errors are 0.0180° and 0.0191°, respectively. The root mean square errors of the angular measurement are 0.012704° and 0.013882°. Since the maximum optical scanning angle is derived from the nonlinear scanning angle transformation function, it is evident that the maximum measurement error depends primarily on the accuracy of this function.

#### Adaptability and stability

The stability and adaptability of the APS are demonstrated by its ability to track and measure both constant and gradually changing scanning angles. Adaptability refers to accurately tracking changing scanning angles despite external disturbances that cause deviations from the target scanning angle. Stability ensures that the sensor maintains high accuracy during long-term operation.

To demonstrate the adaptability and stability, we applied driving voltages with consistent amplitudes and decreasing frequencies to the *x*-axis and the same amplitudes and frequencies to the *y*-axis. We conducted repeated experiments, similar to the accuracy experiments, at ten-minute intervals while maintaining continuous scanning of the M-SM. A total of 8 experiments were performed over 80 min, and the sensing accuracy is presented in Table 2 and Table 3. It was observed that the field of view (FOV) decreases when the frequency deviates from the resonant frequency of the M-SM in the adaptability verification experiment, while the measurement of the *y*-axis scanning angle exhibits high consistency.

**Table 2:** Sensor adaptability analysis during the optical scanning angle decreasing around the *x*-axis

NO.	Truth of FOV (°)	Measured FOV (°)	e_RMS_ (°)	e_max_ (°)
1	10.05392	10.01720	0.0130	0.0183
2	9.69594	9.64813	0.0169	0.0239
3	9.44361	9.46764	0.0085	0.0120
4	9.06353	9.03671	0.0095	0.0134

**Table 3:** Sensor stability analysis during the optical scanning angle holding around the *y*-axis

NO.	Truth of FOV (°)	Measured FOV (°)	e_RMS_ (°)	e_max_ (°)
1	3.90309	3.87033	0.0119	0.0163
2		3.92605	0.0083	0.0115
3		3.87741	0.0093	0.0128
4		3.87627	0.0098	0.0134

For the gradual decrease in the true optical scanning angle of the M-SM around the *x*-axis, from 10.05° to 9.06°, the root mean square error ranged from 0.0085° to 0.0169°, with a maximum error ranging from 0.0120° to 0.0239°. In the case of the M-SM scanned at a constant angle around the *y*-axis, the root mean square error ranged from 0.0083° to 0.0119°, with a maximum error ranging from 0.0115° to 0.0163°.

#### Data update rate

To evaluate the real-time performance of the sensor, the data update rate can be calculated using the following formula:

18 $${S}_{\max ,i}=2{f}_{i}\frac{{\theta }_{m,i}}{{e}_{RMS,i}}$$

where *i* = *x* or *y* indicates that the M-SM is scanned around the *x-* and *y*-axes. S_max,i_ represents the maximum number of data updates per second, while *f*_i_ denotes the resonant frequencies. e_RMS,i_ indicates the root mean square error. The parameters are presented in Table 4.

**Table 4:** Parameters of the M-SM system

Parameter	*x*-axis	*y*-axis
*f* (Hz)	892.0	959.2
*θ*_m_ (°)	10.49	3.90
e_RMS_ (°)	0.01270	0.01388
*S*_max_ (kHz)	1473.6	539.4
*S*_original_ (kHz)	2.2	2.2
*Scale* (1)	669.8	245.2

The data update rate has significantly increased to 1473.6 kHz and 539.4 kHz, corresponding to a 669-fold and 245-fold increase, respectively. This notable improvement effectively resolves the issue of insufficient sampling frequency of capacitive APSs in the kilohertz operating frequency band application.

#### Other specifications

**Fig. 11:**
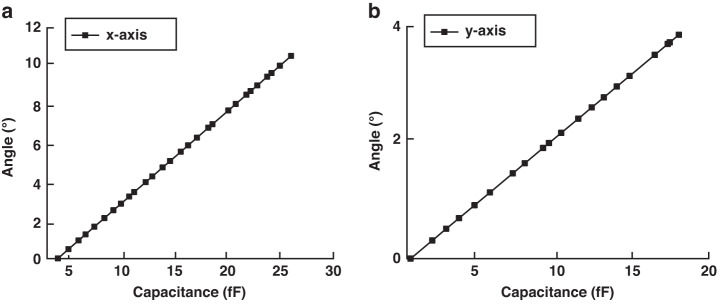
Analysis of the sensor specifications. **a** Sensitivity analysis of *x*-axis optical scanning angles. **b** Sensitivity analysis of *y*-axis optical scanning angles

A drive frequency of 892.0 Hz was chosen to ensure the alignment of the scanning light in a coaxial manner with the x-axis. Upon activation of the APS, we diligently recorded pivotal parameters, whereby the maximum optical scanning angle registered at 10.49°, and the corresponding peak differential capacitance reached 25.57 fF. Subsequently, we conducted experiments to monitor the optical scanning angle and differential capacitance while progressively reducing the drive current until the scanning mirror attained a state of rest. These observations are graphically depicted in Fig. 11a for comprehensive visualization and reference. It is important to note that in the stationary state, the differential capacitance does not reach zero, primarily because of inherent factors such as processing, assembly, and various sources of error.

In a subsequent experimental phase, we adjusted the drive frequency to 959.2 Hz and conducted analogous procedures. The resultant relationship between the optical scanning angle along the y-axis and the corresponding differential capacitance is meticulously illustrated in Fig. 11b. The sensor specifications are shown in Table 5:

**Table 5:** Sensor specifications

Specifications	*x*-axis	*y*-axis
Linearity (%)	99.64	99.36
Nonlinear error (°)	0.0099	0.0086
Sensitivity (°/fF)	215.46	107.45
Resolution (°)	0.0190	0.0094

The nonlinear error remains well below the optimal accuracy attainable through the proposed method. Consequently, it is reasonable to posit the existence of a robust linear relationship between the scanning angle and the corresponding differential capacitance.

The resolution of the system hinges on the inherent resolution of the PCAP01 capacitive-to-digital converter chip, which stands at an impressive 88 aF (where 1 aF equals 1e-3 fF or 1e-6 pF) when operating at a sample rate of 2.2 kHz.

## Materials and methods

### Comparison of different interpolation extrapolation methods

**Fig. 12:**
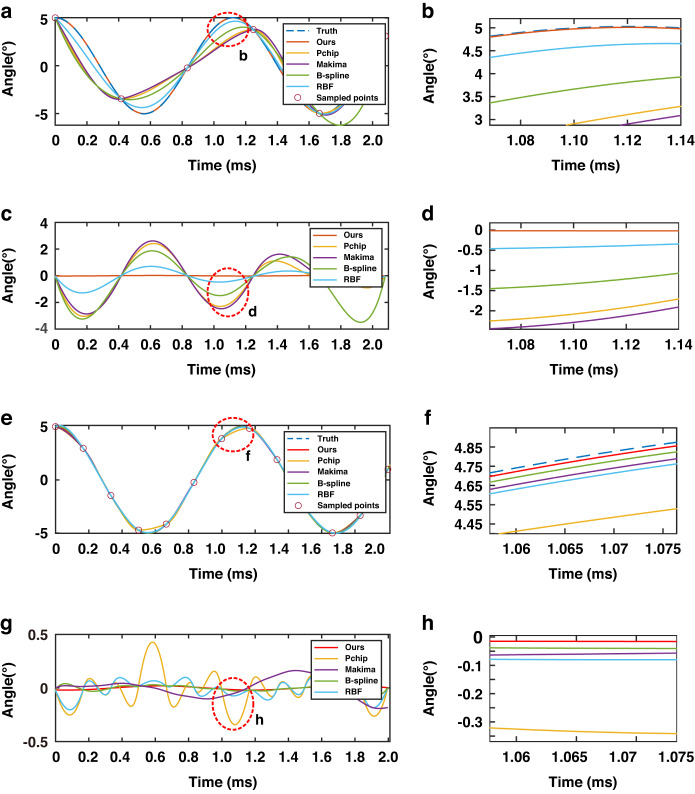
Comparison of the different interpolation extrapolation methods. **a** Comparison of the five interpolation extrapolation methods with angular truth values for Scenario 1 (6 sampling points). **b** An enlarged view centered at approximately 1.1 ms from Fig. 12a. **c** Error curves for five interpolation extrapolation methods. **d** Enlarged view centered at approximately 1.1 ms from Fig. 12c. **e** Comparison of five interpolation extrapolation methods with angular truth values for Case 1 (13 sampling points). **f** Enlarged view centered at approximately 1.065 ms from Fig. 12e. **g** Error curves for five interpolation extrapolation methods. **h** An enlarged view centered at approximately 1.065 ms from Fig. 12g

A comprehensive comparative analysis of the proposed method has been conducted in two distinct scenarios. In the first scenario, six data points were sampled over approximately two cycles of the angular signal, utilizing the existing hardware configuration. The second scenario involved the more challenging task of acquiring 13 sampling points, which was challenging to achieve given the hardware constraints. The visual representation of this comparison is presented in Fig. 12, while the summarized results of this comparative assessment can be found in Table 6.

**Table 6:** Comparison of the Accuracies

Method	Ours(°)	Pchip(°)	Makima(°)	B-spline(°)	RBF(°)
Case 1	0.0139	1.5028	1.5733	1.7367	0.5232
Case 2	0.0142	0.1593	0.0838	0.02447	0.0896

In Scenario 1, the root mean square error (RMSE) associated with the proposed interpolation extrapolation method, leveraging known angular motion characteristics models, represents a mere 2.656% of the minimum error observed with the conventional method (RBF method).

In Scenario 2, characterized by a sampling frequency twice as high as the maximum sampling frequency accommodated by the existing hardware configuration, the RMSE attributed to the proposed method is 58.030% of the minimum error encountered with the conventional method (B-spline method). This outcome underscores the exceptional interpolation and extrapolation accuracy achieved by the proposed method, reaffirming its superior performance in this context.

### Noise analysis

**Fig. 13:**
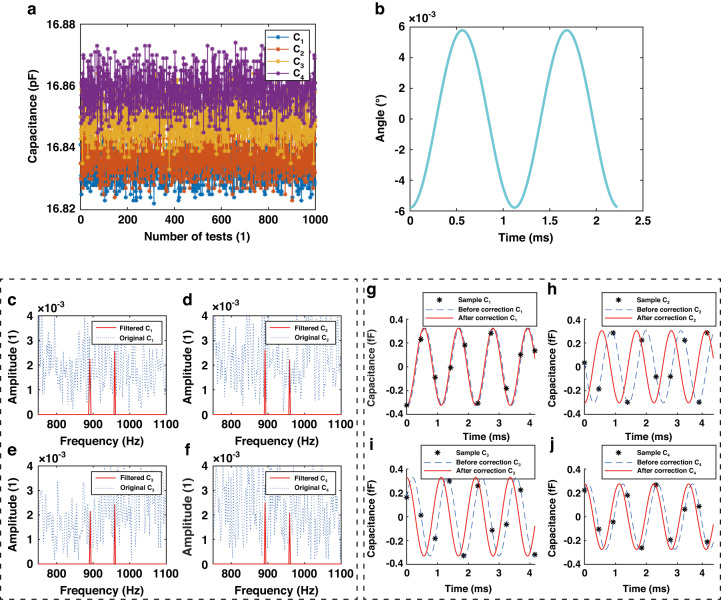
Analysis of noise. **a** Raw noise signal for 4 channels. **b** Noise based on differential calculations. **c**–**f** Amplitude-frequency characteristics curve of the 4-channel noise signal. **g**–**j** 4-Channel noise recovered from the characteristic curve

In the context of our experimentation, when the scanning mirror was at rest, we meticulously collected 1000 sets of data from the 4-channel capacitor, resulting in the acquisition of a raw noise signal, as visually represented in Fig. 13a. Subsequently, we subjected the 4-channel noise signal to digital filtering with a bandpass frequency focusing on a single peak frequency (892 Hz, x-axis). Afterward, a differential calculation was performed, yielding the measurement noise curve for the x-axis optical scanning angle, as depicted in Fig. 13b. It is noteworthy that the maximum noise recorded in this analysis amounts to 0.00578°. Importantly, this value falls below both the measurement accuracy threshold of 0.014° and the system’s resolution of 0.019°. This observation leads us to conclude that the noise level associated with the proposed sensor remains within acceptable tolerances, exerting minimal influence on accuracy and resolution.

We further investigated the amplitude-frequency characteristics of the 4-channel noise signals, which are visually presented in Fig. 13c–f. Following the digital filtering process, the filtered 4-channel noise signals are displayed in Fig. 13g–j. Notably, a significant portion of the noise errors are effectively canceled out subsequent to the phase correction and differential calculation procedures.

Moreover, when we extended this analysis to include scanned rays in the y-axis direction, similar digital filtering methods were applied to eliminate noise signals other than the single-peak frequency of 959.2 Hz. While detailed illustrations of this process are regrettably omitted here due to space constraints, it is paramount to note that the maximum noise observed in this scenario amounts to 0.00672°, which, once again, falls within a tolerable range of influence on the sensor’s performance.

### Measurement uncertainty analysis

In our experiment, the measured optical scanning angles were determined to be 10.5318° (*x*-axis) and 3.8670° (*y*-axis). To quantify the measurement uncertainty associated with this method, the following calculation can be employed.

19 $$\varDelta {\theta }_{x}=\sqrt{{\left(\frac{\partial {\theta }_{x}}{\partial D{C}_{x}}\varDelta D{C}_{x}\right)}^{2}+{\left(\frac{\partial {\theta }_{x}}{\partial \omega }\varDelta \omega\right)}^{2}+{\left(\frac{\partial {\theta }_{x}}{\partial h}\varDelta h\right)}^{2}+{\varDelta }_{extra}^{2}}$$

20 $$\varDelta {\theta }_{y}=\sqrt{{\left(\frac{\partial {\theta }_{y}}{\partial D{C}_{y}}\varDelta D{C}_{y}\right)}^{2}+{\left(\frac{\partial {\theta }_{y}}{\partial l}\varDelta l\right)}^{2}+{\left(\frac{\partial {\theta }_{y}}{\partial h}\varDelta h\right)}^{2}+{\varDelta }_{extra}^{2}}$$

21 $${\theta }_{x}(t)={f}_{x}(D{C}_{x},w,h)$$

Equation (21) is the inverse function of Eq. (5).

22 $${\theta }_{y}(t)={f}_{y}(D{C}_{y},l,h)$$

Equation (22) represents the inverse function of Eq. (7). The uncertainties associated with the measurements are as follows: ∆*DC*_x_ = ∆*DC*_y_ = 0.001 pF, indicating the measurement uncertainty of the capacitance-to-digital circuit; ∆*w* = ∆*l* = 0.001 mm, representing the fabrication uncertainty; and ∆*h* = 0.1 mm, reflecting the assembling uncertainty. Additionally, ∆_extra_ ≈ 0.004° accounts for the edge effect error caused by the leakage of the edge capacitance^25^. By substituting all the aforementioned uncertainty terms into Eqs. (9) and (10), the angular measurement uncertainties along the *x*-axis and *y*-axis are calculated as ∆*θ*_x_ = ∆*θ*_y_ = 0.0105°.

Equation (11) provides the true optical scanning angle θ_PSD_ of the M-SM, which is utilized to calculate the measurement uncertainty of the PSD-based angular measurement method.

23 $${\theta }_{PSD}=\frac{360}{\pi }arc\,\tan \frac{{V}_{p}}{450\cdot d}$$

The distance between the M-SM and the PSD screen, denoted as *d*, is 60 mm. When the laser was scanned on the PSD screen, the scanning distance was determined by the peak-to-peak voltage *V*_p_. In this experiment, the peak-to-peak voltages were measured as 2.48 V and 0.92 V, corresponding to the true optical scan angles of 10.4960° and 3.9031°, respectively.

The measurement uncertainty of the PSD-based method was determined using the following calculation:

24 $$\varDelta {\theta }_{PSD}=\sqrt{{\left(\frac{\partial {\theta }_{PSD}}{\partial {V}_{p}}\varDelta {V}_{p}\right)}^{2}+{\left(\frac{\partial {\theta }_{PSD}}{\partial d}\varDelta d\right)}^{2}}$$

where the voltage measurement uncertainty, denoted as ∆*V*_p_, depends on the device used for measuring *V*_p_. In this experiment, an ordinary oscilloscope was employed, resulting in a voltage measurement uncertainty of 0.2 mV. On the other hand, the distance measurement uncertainty, represented by ∆*d*, relies on the tool used to measure the distance between the M-SM and PSD screen. A spiral micrometer was utilized, yielding a measurement uncertainty of 0.001 mm. By substituting the aforementioned uncertainty terms into Eq. (12), the PSD-based angular measurement uncertainty was determined to be ∆*θ*_PSD_ = 0.00113°.

Since the measurement uncertainty of the PSD-based method was approximately 10 times smaller than that of the capacitive-based method, *θ*_PSD_ can be regarded as a reliable representation of the true optical scanning angle.

### Quantitative comparisons in different unmanned scenarios

To underscore the practical utility of the proposed sensors, it is imperative to conduct quantitative comparisons across different usage scenarios. In pursuit of this objective, we have established two distinct scenarios:

#### Scenario 1: Flat roadway

In this setting, the frequency and amplitude of the scanning mirror’s movement remain unchanged. The proposed sensor operates in a continuous capture mode, wherein it continuously provides feedback on the capacitive signal and compares it with the initial capacitive feedback signal. If the disparity between the two signals falls below a predefined threshold value set by the system, the capture mode is sustained.

#### Scenario 2: Bumpy roadway

In Scenario 2, the sensor encounters perturbations due to the uneven terrain, which, in turn, influence the scanning angle of the miniaturized scanning mirror (M-SM). To effectively analyze these dynamic changes, the comprehensive data processing algorithm depicted in Fig. 1c is activated. This algorithm continuously operates to update the motion feature model, enabling the sensor to adapt seamlessly to fluctuations in the scanning angle induced by the uneven road surface. This adaptive capability ensures that the sensor can maintain accuracy and functionality even in challenging conditions.

We aim to quantitatively compare the advantages and disadvantages of Scenario 1 and Scenario 2 in four key dimensions: the angular amplitude update rate, response speed, accuracy on a flat road, and accuracy on a bumpy road. The results are shown in Table 7.

**Table 7:** Quantitative comparison between the two scenarios

Scenario	Flat roadway	Bumpy roadway
Angular amplitude update rate	No need	2.227 Hz
Response time	0.11607 ms	449.03 ms
Accuracy on a flat road	0.0139°	0.0139°
Accuracy on a bumpy road	0.288°^35^	0.0169°

Note: The term “Angular Magnitude Update Rate” specifically denotes the frequency at which one complete data processing cycle is executed, encompassing all seven steps outlined in Fig. 1c

In the flat section scenario, the angular amplitude remains constant, and the response time relies on the reciprocal of the maximum sampling rate, which is 8.96 kHz for the CTDC. The accuracy in the flat section aligns with the specifications detailed in Section “Specifications” of the manuscript. For the bumpy road section, the accuracy is contingent on the impact of shocks on the scanning mirror’s angle. In the case of the proposed scanning mirror structure, a shock of 400 g induces an angular change of approximately 0.288°.

In scenarios involving bumpy road sections, the update frequency *f*_*tracking*_ for the angular magnitude is determined by considering the cumulative time needed for FFT filtering, signal recovery, and the sampling process. The calculation process for *f*_*tracking*_ is detailed in Eq. 26:

26 $${f}_{tracking}=\frac{1}{{t}_{FFT}+{t}_{sample}}=2.227Hz$$

where tFFT represents the time required to perform the FFT on 4000 capacitances. When utilizing the STM32F407 microcontroller with the floating-point processing unit enabled, the FFT computation time, tDFT, is set to approximately 2563.90 microseconds. The response time is equal to the reciprocal of *f*_*tracking*_, the accuracy of the flat section is equal to the accuracy of the “Accuracy” section in Section “Specifications”, and the accuracy of the bumpy section is equal to the accuracy of the “Adaptability and stability” in Section “Specifications”.

## Discussion

Capacitive sensors offer high accuracy capabilities; however, their low sampling frequency hinders direct utilization in M-SM-based LiDAR imaging applications. To address this limitation, the proposed method bridges the gap in angle measurement by employing the capacitive principle of M-SM, thereby ensuring imaging accuracy in M-SM-based LiDAR systems. A novel set of data processing algorithms is introduced to address the issue of insufficient sampling frequencies. Additionally, these algorithms enhance the measurement range of scanning angles and effectively resolve the problem of asynchronous communication that causes confusion in angular signal timestamps.

In terms of the angular measurement accuracy, the highest precision method currently available is a piezoresistive sensor based on UKF filtering. When compared to our proposed method, this sensor demonstrates significantly improved accuracy, thus highlighting the superior performance of capacitive sensors in angular measurement. Furthermore, the proposed data processing method introduces greater flexibility in terms of software and can be applied to any angular measurement method that satisfies the motion characteristics model, extending beyond exclusive reliance on the sampling circuit for angular information.

To meet the progressively increasing resolution requirements of LiDAR, the sampling rate of the angle sensor must be correspondingly increased. However, hardware limitations constrain the extent to which the sampling rate can be improved. Therefore, a reasonable combination with the angular motion characteristics model becomes an effective approach for balancing the relationship between high sampling frequency and low cost. The introduction of a 2-D nonlinear angular transformation function imposes a universal motion constraint on the M-SM. This is achieved through calculations involving the free-position parallel plate capacitor in space, enabling the establishment of an accurate, reliable, and universal angular transformation equation that has been experimentally proven to further enhance accuracy.

Although the motion characteristics model can enhance the sampling frequency, the update rate of the model itself is low. Consequently, if the motion state of the M-SM changes during operation but is not updated in a timely manner, it may adversely affect accuracy. To address this issue, when faced with anomalies in the motion state of the M-SM, the number of capacitances can be reduced for the application of the FFT algorithm, or the number of CTDC can be increased. These measures effectively multiply the update speed of the motion model and enhance the proposed sensor’s resilience to extreme environments. Exploring additional methods to improve the update rate of the model can further enhance the performance of the data processing method.

## Data Availability

The datasets generated during the current study are available from the corresponding author upon reasonable request.
